# Utility of endoscopic ultrasonography-guided fine-needle biopsy (EUS-FNB) for diagnosing small subepithelial lesions (< 20 mm)

**DOI:** 10.1007/s40477-020-00548-6

**Published:** 2021-01-29

**Authors:** Masanari Sekine, Takaya Miura, Junichi Fujiwara, Takeshi Uehara, Takeharu Asano, Satohiro Matsumoto, Hiroyuki Miyatani, Hirosato Mashima

**Affiliations:** Department of Gastroenterology, Saitama Medical Center, Jichi Medical University, 1-847 Amanuma-cho, Omiya-ku, Saitama City, Saitama 330-8503 Japan

**Keywords:** Endoscopic ultrasonography, Subepithelial lesions, Endoscopic ultrasound-guided fine-needle biopsy, Gastrointestinal stromal tumor

## Abstract

**Aim:**

Subepithelial lesions (SELs) are defined as being located under the mucosa. Presently, endoscopic ultrasound-guided fine-needle aspiration (EUS-FNA) is commonly performed to diagnose SELs. With the development of new puncture needles, endoscopic ultrasound-guided fine-needle biopsy (EUS-FNB), which allows for the acquisition of large tissue samples, has been proposed. However, studies on EUS-FNB of SELs measuring < 20 mm have not yielded satisfactory results. Therefore, we aimed to assess the performance and usefulness of EUS-FNB of SELs measuring less than < 20 mm.

**Methods:**

The present study included 62 patients who underwent EUS-FNA or EUS-FNB for SELs at our hospital between January 2015 and March 2019. EUS-FNA was performed using fine-needle aspiration needles, and EUS-FNB was performed using fine-needle biopsy needles. These needles, which come in different shapes and diameters, were compared in terms of their usefulness in performing procedures for SELs measuring ≥ 20 mm and those measuring < 20 mm.

**Results:**

For SELs measuring ≥ 20 mm, the use of needles with a large diameter, such as 19 or 20 G, resulted in significantly improved diagnostic rates. For SELs measuring < 20 mm, the use of FNB needles showed significantly improved diagnostic rates, regardless of the size of the puncture needles.

**Conclusion:**

Even when SELs are less than 20 mm, they might have malignant potential, and histological diagnosis may be desirable in some cases. EUS-FNB has an advantage over EUS-FNA in the diagnosis of SELs measuring < 20 mm.

## Introduction

Subepithelial lesions (SELs) are defined as being located under the mucosa. They develop throughout the gastrointestinal tract, and include a wide spectrum of lesions, such as lipoma, heterotopic pancreas, leiomyoma, schwannoma, gastrointestinal stromal tumor (GIST), leiomyosarcoma, and neuroendocrine neoplasm. EUS (endoscopic ultrasonography) and/or UMP (ultrasonic microprobe) are important for the diagnosis, because of the location of SELs. The malignant risk of GIST is evaluated by the imaging findings and the risk classification. Imaging findings of GIST suggesting malignancy included an ulceration on the top of an SEL in conventional esophagogastroduodenoscopy, and irregular outline, calcification, cystic change, and heterogeneous echo texture on EUS images. The risk classification criteria is based on the tumor size, localization, and mitotic activity [1–3].

The Japanese GIST guidelines state that SELs measuring ≤ 20 mm should be monitored without treatment. However, we have experienced a case of GIST that increased from 18 to 84 mm over 2 years and caused liver metastasis [4]. Therefore, in some cases, it is necessary to diagnose SELs, even those measuring < 20 mm, to determine appropriate treatment strategies in some cases. Diagnostic procedures, such as boring biopsy and mucosal incision-assisted biopsy, have high diagnostic accuracy [5]. Presently, endoscopic ultrasound-guided fine-needle aspiration (EUS-FNA) is commonly performed. With the development of new puncture needles, EUS-guided fine needle biopsy (EUS-FNB), which allows for the acquisition of larger tissue samples, has been reported to have a high diagnostic accuracy [6, 7]. However, studies on EUS-FNB of SELs measuring < 20 mm have not yielded satisfactory results. Regarding the usefulness of EUS-FNB for < 20 mm, Hedenström [8] report beneficial, while Fujita [9] report as contrary. Therefore, in this study, we aimed to assesses the performance and usefulness of EUS-FNB of SELs measuring < 20 mm.

## Material and methods

### Study design

This was a pilot, retrospective, single-center study.

### Patients

The present study included 62 patients who underwent EUS-FNA or EUS-FNB of SELs at Saitama Medical Center, Jichi Medical University between January 2015 and March 2019. Of these patients, 24 had SELs measuring < 20 mm.

### EUS-FNA and EUS-FNB

The EUS-FNA procedure was performed by experienced endosonographers. A convex linear-array echoendoscope (GF-UCT260; Olympus Optical Corp, Tokyo, Japan) connected to an ultrasonography platform (ME-2 Premier Plus; Olympus Optical Corp, Tokyo, Japan) was used in this procedure. EUS was performed under conscious sedation using intravenous midazolam and pethidine hydrochloride.

**Fig. 1:**
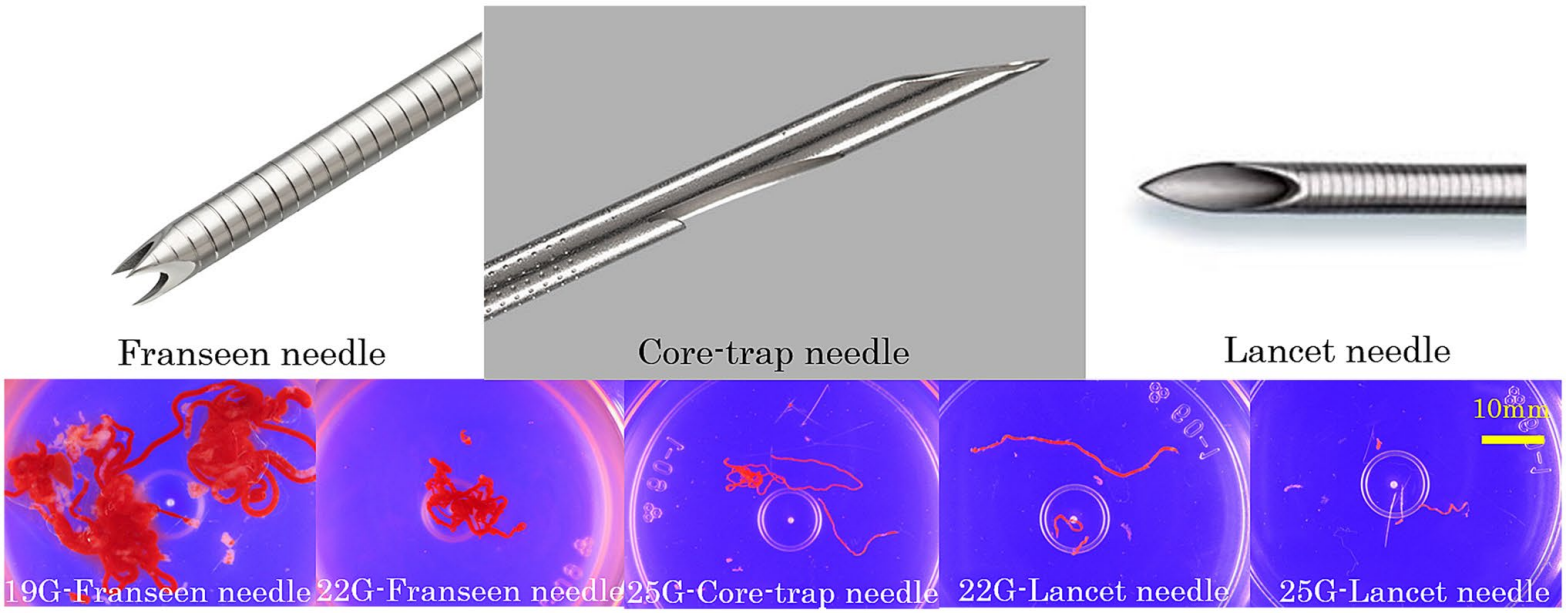
The shape of the needles and the macroscopic samples acquired

The details of the procedure were as follows: The endosonographers selected a shape of needle arbitrarily (fine needle aspiration [FNA] needle or fine needle biopsy [FNB] needle to obtain specimens for cytological and histological analysis (Fig. 1). The size of the needle was selected based on the size of tumor and the empirical judgement of the endosonographers. Expect^®^ (Boston Scientific Japan, Tokyo, Japan), EZshot3^®^ (Olympus Optical Corp, Tokyo, Japan), and SonoTip^®^ (Medicos Hirata, Osaka, Japan), which are all lancet needles, were used in the FNA group. Acquire_®_ (Boston Scientific Japan) and ProCore_®_ (Wilson-Cook Medical Inc., USA) were used in the FNB group; the former is a Franseen needle and the latter is a core-trap needle. After properly targeting the mass, the FNA or FNB needle was advanced under EUS guidance, and the lesion was punctured with the needle. The EUS-FNA needle was moved back and forth 15 times while suction was applied with a 20 ml syringe, and the needle of EUS-FNB was moved back and forth 15 times without suction. A maximum of three punctures was performed with both needles.

### Histological evaluation

After the procedure, specimens were prepared for cytological studies using Papanicolaou staining. Samples were also exposed to 10% formalin, then processed as a tissue block for histopathological evaluation using hematoxylin–eosin and IHC staining. GIST was diagnosed by positive c-kit staining with or without positive CD34 staining, Leiomyoma by positive desmin staining, and Schwannoma by positive S-100 staining.

The final diagnosis was made by examining surgical samples from patients undergoing surgery, biopsy samples from patients undergoing endoscopic biopsy procedures, or EUS-FNA or EUS-FNB samples. When EUS-FNA or EUS-FNB samples revealed no malignant findings, patients were monitored without treatment, and lesions showing neither growth nor other changes were determined to be benign.

### Statistical analysis

All values represent the mean ± standard deviation. A bivariate analysis was performed using the Chi-square test for categorical variables. *P* values of < 0.05 were considered significant, and all p-values were two-sided. Data were analyzed using EZR version 1.41 statistical software (Saitama Medical Center, Jichi Medical University, Saitama, Japan) [10].

## Results

### Patient cohort

Table 1 shows the background patient characteristics, tumor size, and EUS-FNA or -FNB procedures. Except for tumor size, there were no significant differences between the group with SELs measuring < 20 mm and the group with SELs measuring ≥ 20 mm.

**Table 1:** Characteristics of the patients, tumor size, and procedures

The number of cases	All (62)	< 20 mm (24)	> 20 mm (38)	*P* value
The average age (years. range)	63.2 (37–86)	62.8 (38–86)	63.7 (37–85)	N.S
Male: Female	25:37	11:13	14:24	N.S
none: antiplatelet drugs: anticoagulant	58:2:2	23:1:0	35:1:2	N.S
The size of the puncture needles (19G:20G:22G:25G)	5:34:11:12	3:10:3:8	2:24:8:4	N.S
The puncture site (Esophagus: Stomach: Duodenum: Rectum)	1:49:8:4	0:19:4:1	1:30:4:3	N.S
The number of puncture (times, range)	1.6 (1–3)	1.8 (1–3)	1.9(1–3)	N.S
The average size of tumor (mm, range)	31.7 (10–264)	15.8 (10–19)	41.8 (20–264)	0.004
Complication	1	0	1	N.S
			N.S.: not significant	

### The final diagnosis and comparison of the diagnostic rate

The final diagnosis was made from surgical samples in 29 cases, EUS-FNA or EUS-FNB samples in 28 cases, an endoscopic biopsy sample in 1 case, and the clinical courses in 4 cases. The endoscopic biopsy sample was taken from the depressed lesion on the top of SEL, and the EUS-FNA sample showed no conclusive data.

Table 2 shows the number of patients with SELs measuring < 20 mm and ≥ 20 mm, along with the diagnostic rate in terms of the final diagnoses in all patients. The most common final diagnosis was GIST, which was detected in 30 patients, with a diagnostic rate of 90%. This was followed by nerve sheath tumors, which were detected in 7 patients, with a diagnostic rate of 85.7%, and leiomyoma, which was detected in 6 patients, with a diagnostic rate of 100%. The diagnostic rate for GISTs was 100% in patients with SELs measuring < 20 mm and 85.7% in those with SELs measuring ≥ 20 mm; the diagnostic rates for Schwannoma in patients with SELs measuring < 20 mm and in those with SELs measuring ≥ 20 mm were 100% and 80.0%, respectively.

**Table 2:** The final diagnosis and the diagnostic rate

The final diagnosis	The diagnostic rate of all (62)	The diagnostic rate of < 20 mm (24)	The diagnostic rate of > 20 mm (38)
GIST	90% (27/30)	100% (9/9)	85.7% (18/21)
Schwannoma	85.7% (6/7)	100% (2/2)	80.0% (4/5)
Leiomyoma	100% (6/6)	100% (6/6)	–
Gastric cancer	100% (4/4)	–	100% (4/4)
NEN	66.7% (2/3)	100% (2/2)	0% (0/1)
Brunner’s glands	66.7% (2/3)	100% (2/2)	0% (0/1)
Ectopic pancreas	50% (1/2)	0% (0/1)	100% (1/1)
Leiomyosarcoma	100% (1/1)	–	100% (1/1)
Sarcoidosis	0% (0/1)	0% (0/1)	–
Benign (Inflammatory change, etc.)	100% (5/5)	100% (1/1)	100% (4/4)

### Comparison of the diagnostic rate by factors

Table 3 shows the diagnostic rate based on the size of the tumor and the shape and size of the puncture needles. It also shows the details of the puncture site. There was no bias in the distribution of target organs. Bold data in Table 3 indicates success rate that did not reach 100% in the puncture site and needle. We then compared the diagnostic rates of the FNB group and FNA group for SELs measuring < 20 mm and those measuring ≥ 20 mm (Table 4A). For SELs measuring < 20 mm, the diagnostic rate in the FNB group (100%) was significantly higher than that in the FNA group (72.7%, *p* = 0.025), whereas no significant difference was observed in the diagnosis of SELs measuring ≥ 20 mm (FNB group 77.7% vs FNA group 75.0%, *p* = 0.606). We next compared the diagnostic rate between the sizes of the puncture needles (19 G + 20 G and 22 G + 25 G, Table 4B). For SELs measuring < 20 mm, the sizes of the puncture needles were not important to the diagnostic rate (19G + 20G 90.9% vs 22G + 25G 84.6%, *p* = 0.442), whereas for SELs measuring ≥ 20 mm, the diagnostic rate was significantly higher for needles with a large diameter (19 G + 20 G 83.3%) than for needles with a small diameter (22G + 25G 73.1%, *p* = 0.010).

**Table 3:** The diagnostic rate according to the tumor size and the shape and size of the puncture needles, and the distribution of target organ

Tumor size	The shape of the puncture needles	The size of the puncture needles	The diagnostic rate	The details of the puncture site							
				Esophagus	Stomach				Duodenum		Rectum
					Fornix	Cardia	Body	Antrum	Bulb	2nd	
< 20 mm	EUS-FNB	19G	100% (6/6)			3/3	2/2			1/1	
	Franseen needle	20G	100% (3/3)		1/1	1/1	1/1				
	Core-trap needle	22G	100% (1/1)						1/1		
		25G	100% (3/3)			1/1	1/1		1/1		
	EUS-FNA	19G	50% (1/2)							1/1	**0/1**
	Lancet needle	22G	77.8% (7/9)		3/3	1/1	**3/5**				
≥ 20 mm	EUS-FNB	19G	100% (4/4)		1/1		2/2			1/1	
	Franseen needle	20G	75% (6/8)		1/1	1/1	4/4		**0/2**		
	Core-trap needle	22G	80% (4/5)		1/1		**2/3**				1/1
		25G	0% (0/1)							**0/1**	
	EUS-FNA	22G	73.7% (14/19)		2/2	**0/1**	**11/13**	**1/2**			**1/2**
	Lancet needle	25G	100% (1/1)	1/1

**Table 4:** Comparison of the diagnostic rate based on the shape (A) and size (B) of the puncture needles

Tumor size	The shape of the puncture needles	The diagnostic rate	*P* value
*(A)*			
< 20 mm	EUS-FNB	100% (13/13)	0.025
	Franseen needle		
	Core-trap needle		
	EUS-FNA	72.7% (8/11)	
	Lancet needle		
≥ 20 mm	EUS-FNB	77.7% (14/18)	0.606
	Franseen needle		
	Core-trap needle		
	EUS-FNA	75.0% (15/20)	
	Lancet needle		
*(B)*			
< 20 mm	19G + 20G	90.9% (10/11)	0.442
	22G + 25G	84.6% (11/13)	
≥ 20 mm	19G + 20G	83.3% (10/12)	0.01
	22G + 25G	73.1% (19/26)	

## Discussion

SELs are sometimes incidentally found during esophagogastroduodenoscopy or colonoscopy. The diagnosis and treatment are based on the Japanese GIST guidelines. The guidelines states that SELs measuring 20 mm to less than 50 mm should be resected when they demonstrate clinical symptoms or malignant findings and that surgery is indicated for those that are diagnosed as GIST by pathology. SELs measuring < 20 mm and without malignant findings should be monitored without treatment. EUS and UMP are important for the diagnostic imaging of SELs, and EUS has the advantage of tissue sampling at the same time. EUS-FNA was covered by the Japanese National Health Insurance Scheme in 2010 and has been widely accepted since then. EUS-FNA is a minimally invasive and an extremely useful procedure for diagnosing SELs. Its diagnostic rate ranges from 70 to 92% [11–13]. In this study, the diagnostic rate was 86.0% (49/57 patients, excluding benign disease).

At present, the lancet needle is generally used. The reliable acquisition of lesion samples is important for diagnosing SELs. In the case of GISTs, karyokinetic figures and immunostaining (Ki-67 labeling index) are important to assess the risk of growth and metastasis. Thus, the acquisition of large tissue samples is ideal. In recent years, core-trap and Franseen needles (EUS-FNB needles) have been developed. Many reports have indicated that EUS-FNB is more useful than EUS-FNA [6, 7]. However, many such studies restricted their subjects to those with SELs measuring ≥ 20 mm. There are reports indicating that EUS-FNB is not useful for SELs measuring < 20 mm [9, 14–16].

However, Akahoshi et al. reported that 23% of 43 surgical patients with SELs measuring < 20 mm showed a moderate risk, based on the modified Fletcher criteria [17], and we have reported cases of SELs measuring < 20 mm that grew rapidly [4]. SELs measuring < 20 mm and showing malignant findings should be diagnosed and evaluated for the risk of metastasis.

The present study showed that the use of needles with large diameters, such as 19 or 20 G, improved diagnostic rate for SELs measuring ≥ 20 mm. This result was consistent with those of the previous study [18]. For SELs measuring < 20 mm, the diagnostic rate of EUS-FNB was higher than that of EUS-FNA, regardless of needle size. In other words, FNB needles are more useful for SELs measuring < 20 mm. In the case of small lesions located at sites difficult to puncture, needles with a small diameter are often required to ensure a successful puncture. In such a situation, EUS-FNB needles having an excellent tissue acquisition rate are recommended and can be used with a proper technique. For example, when the lesion is located at the greater curvature of the stomach, the needle tip tends to bounce off, and this makes it difficult to puncture the lesion. In such a case, we thrust the needle into the lesion with a single, short, and rapid movement, so we can get a richer tissue sample.

The limitations of the present study include the retrospective study design, the single-center nature, the small sample size, and the arbitrary choice of the needle. Only a few reports have indicated that EUS-FNB is useful for SELs measuring < 20 mm. Therefore, the findings from our study will contribute to the literature.

## Conclusion

Even when SELs are less than 20 mm in diameter, those with malignant findings should be diagnosed histologically. When we need to diagnose SELs measuring < 20 mm, choosing an appropriate size of EUS-FNB needle is important.

## Ethical statement

The study was approved by the ethical committee of Saitama Medical Center, Jichi Medical University (Register Code: S18-115), and conducted in accordance with the Declaration of Helsinki.
